# Supplementary material for: Medication Intake as a Factor for Non-Initiation and Cessation of Breastfeeding: A Prospective Cohort Study in Greece during the COVID-19 Pandemic
Source: Children (Basel). 2023 Mar 18;10(3):586. doi: 10.3390/children10030586 (PMC10047701; doi:10.3390/children10030586)
Supplement: Supplementary file 1 [file children-10-00586-s001.zip › children-2240182-supplementary.pdf]

**Table S1**. Medicines that women used during the postpartum period and show disagreement in risk classification according to Lactmed and Hale. (Attica/Greece 2020; N=847)

| ATC | Medication | Lactmed | Hale | N | % |
|---|---|---|---|---|---|
| J01MA | Ciprofloxacin | Probably Compatible. Avoiding BF for 3-4 hours decreases the exposure. | L3. Use alternative drugs if possible. | 11 | 1.3 |
| J01FF | Clindamycin | Probably Compatible. Use alternative drugs if possible. | L2. LD. Probably Compatible. | 4 | 0.5 |
| N02AB | Meperidine | LD. Infant's sedation. Use alternative drugs. | L4 | 7 | 0.8 |
| J01XD | Metronidazole | Opinions vary. Avoid long term therapy. Avoid BF for 12-24h after last maternal dose. Use alternative drugs if possible. | L2 | 224 | 26.4 |
| J01XE | Nitrofurantoin | Avoid BF when infant <8 days or G6PD deficiency. Use alternative drugs. | L2. Avoid BF when infant <1 month or G6PD deficiency. | 2 | 0.2 |
| A02BA | Ranitidine | Use alternative drugs. Ranitidine spontaneously breaks down to a cancer-causing chemical. | L2 | 7 | 0.8 |
| N02CC | Sumatriptan | LD. Probably Compatible. Withholding breastfeeding in preterm infants might be helpful. | L3 | 2 | 0.2 |
| N03AG | Valproic Acid | Probably Compatible. Monitor infant for side effects. | L4. Neurobehavioral complications. | 2 | 0.2 |

BF: Breastfeeding, LD: Limited Data.

**Table S2.** Risk classification of medicines used during the postpartum period according to Lactmed and Hale.(Attica/Greece 2020; N=847).

| ATC | Medication | Lactmed | Hale | N | % |
|---|---|---|---|---|---|
| B01AC | Acetylosalicylic Acid | Probably compatible (lowdose). | L2 | 7 | 0.8 |
| J05AB | Acyclovir | Compatible. | L2 | 1 | 0.1 |
| L04AB | Adalimubab | LD. Probably compatible. | L3 | 4 | 0.5 |
| L04AA | Alemtuzumab | ND. Use with caution or avoid, especially while nursing a newborn or preterm infant. | L4 | 1 | 0.1 |
| G04CA | Alfuzosin | Not classified. | L4 | 1 | 0.1 |
| N05BA | Alprazolam | LD. Probably Compatible in low dose and short-term use. Use alternative drugs. | L3 | 2 | 0.2 |
| A02AD | Aluminium Hydroxide & Magnesium Hydroxide | AH: Not classified.<br>MH: Compatible. | AH: Not classified.<br>MH: L1 | 2 | 0.2 |
| R05CB | Ambroxol | Not classified. | Not classified. | 2 | 0.2 |
| J01MB | Amikacin | Probably compatible. | L2 | 6 | 0.7 |
| C08CA | Amlodipine | LD. Probably compatible. | L3 | 1 | 0.1 |
| J01CA | Amoxicillin | LD. Compatible. | L1 | 14 | 1.7 |
| J01CAR | Amoxicillin & Clavulanic Acid | LD. Compatible. | L1 | 10 | 1.2 |
| J01CA | Ampicillin | Compatible. | L1 | 7 | 0.8 |
| N05AX | Aripiprazole | LD. Probably Compatible in low doses. Use alternative drugs if possible. May suppress lactation. | L3. May suppress lactation. | 2 | 0.2 |
| C10AA | Atorvastatin | ND. Use alternative drugs. | L3 | 1 | 0.1 |
| L04AX | Azathioprine | Probably Compatible. Use with caution during BF. Avoiding BF for 4 hours markedly decreases drug levels in breast milk. | L3. Use with caution. Monitor infants for signs of immunosuppression. | 3 | 0.4 |
| J01FA | Azithromycin | Probably Compatible. Risk for infantile hypertrophic pyloric stenosis (?). | L2 | 4 | 0.5 |
| D07CC | Betamethasone &Gentamycine (Topical) | Compatible for short-term topical use. Avoid nipple area. | L3 | 2 | 0.2 |
| N05BA | Bromazepam | Not classified. | Not classified. | 2 | 0.2 |
| R05CB | Bromhexine | Not classified. | Not classified. | 2 | 0.2 |
| R01AD | Budesonide | Compatible. | L1 | 2 | 0.2 |
| N07BC V03AB | Buprenorphine & Naloxone | LD. Probably Compatible. | L2 | 1 | 0.1 |
| A03D | Butylscopolamine&Paracetamole | Not classified. | Not classified. | 10 | 1.2 |
| G02CB | Cabergolin | ND. Probably Compatible. Suppresses Lactation – Avoid during lactation (Women treated with cabergoline for pituitary adenomas who become pregnant can breastfeed their infants with no apparent risk of recurrence). | L3. Careful administration in patient with hyperprolactinemia. | 111 | 13.1 |
| A12A | Calcium Salt | Not classified. | L3 | 558 | 65.9 |
| N03AF | Carbamazepine | LD. Probably compatible. | L2 | 1 | 0.1 |
| J01DC | Cefaclor | LD. Compatible. | L1 | 66 | 7.8 |
| J01DC | Cefoxitin | Compatible. | L1 | 239 | 28.2 |

| | | | | | |
|---|---|---|---|---|---|
| J01DD | Ceftriaxone | LD. Compatible. | L1 | 20 | 2.4 |
| J01DC | Cefuroxime | LD. Probably compatible. | L2 | 326 | 38.5 |
| R06AE | Cetirizine | Compatible. High doses may decrease milk supply. | L2 | 1 | 0.1 |
| A02BA | Cimetidine | LD. Probably Compatible for infants >2 months—Use alternative drugs (potential for causing hepatic enzyme inhibition). | L1. Compatible for short-term Use alternative drugs if possible. | 4 | 0.5 |
| J01MA | Ciprofloxacin | Probably Compatible. Avoiding BF for 3-4 hours decreases the exposure. | L3. Use alternative drugs if possible. | 11 | 1.3 |
| N06AB | Citalopram | LD. Probably Compatible. Use alternative drugs if possible. | L2. Use alternative drugs if possible. | 2 | 0.2 |
| J01FA | Clarithromycin | Probably Compatible. Risk for infantile hypertrophic pyloric stenosis (?). | L1 | 7 | 0.8 |
| J01FF | Clindamycin | Probably Compatible. Use alternative drugs if possible. | L2 | 4 | 0.5 |
| N03AE | Clonazepam | Compatible to use cautiously. Use alternative drugs if possible (long half-life). | L3 | 1 | 0.1 |
| M01AB | Diclofenac | LD. Probably Compatible. | L2 | 679 | 80.2 |
| R01AD | Dexamethasone (Topical) | ND. Probably Compatible. | L3 | 3 | 0.4 |
| R06AB | Dimethindene | Not classified. | Not classified. | 3 | 0.4 |
| L04AX | Dimethyl fumarate | LD. Probably Compatible. | L2 | 2 | 0.2 |
| J01AA | Doxycycline | Compatible in short-term use. Avoid prolonged use. | L3. Avoid prolonged use >21 days (dental staining or decrease bone growth). | 1 | 0.1 |
| G03AA | Drospirenone&Ethinylestradiol | Not classified. | L3. May suppress lactation. | 3 | 0.4 |
| G02AB | Ergometrine or Ergonovin Maleate | Avoid during lactation. Lowers PRL, decreases BF rates. | L3. Avoid prolonged use, may suppress lactation. | 559 | 66 |
| J01FA | Erythromycin | Probably Compatible. Risk for infantile hypertrophic pyloric stenosis (?). | L3. Risk for infantile hypertrophic pyloric stenosis (?). | 2 | 0.2 |
| N06AB | Escitalopram | LD. Probably Compatible. | L2 | 5 | 0.6 |
| M01AH | Etoricoxib | Not classified. | Not classified. | 1 | 0.1 |
| N01AH | Fentanyl | LD. Probably Compatible (low dose). | L2 | 631 | 74.5 |
| G01AF | Fenticonazole | Not classified. | Not classified. | 2 | 0.2 |
| B03A | Ferrous Sulfate | Compatible. | L1 | 642 | 75.8 |
| L04AA | Fingolimod | Avoid during lactation. Potential toxicity to the infant. | L5 | 2 | 0.2 |
| H02AA | Fludrocortisone | Not classified. | L3 | 4 | 0.5 |
| N06AB | Fluoxetine | LD. Probably Compatible. Use alternative drugs if possible. | L2. Use alternative drugs if possible. | 1 | 0.1 |
| B03BB | Folic Acid | Not classified. | L1 | 47 | 5.5 |
| V08CA | Gadobutrol | LD. Probably Compatible. Use alternative agents with published experience. | L3 | 1 | 0.1 |
| J01MB | Gentamicin | LD. Probably Compatible. | L2 | 15 | 1.8 |
| S01CA | Gentamicin & Dexamethasone | LD. Probably Compatible. | Not classified. | 2 | 0.2 |
| B01AB | Heparin (low molecular weight) | ND. Probably Compatible. | L2 | 474 | 56 |

| | | | | | |
|---|---|---|---|---|---|
| J06BA | Human Immunoglobulin G | Compatible. | Not classified. | 1 | 0.1 |
| H02AB | Hydrocortisone | ND. Use alternative drugs. | L3. Low dosages, short-term use. | 12 | 1.4 |
| P01BA | Hydroxychloroquine | LD. Probably Compatible. | L2 | 4 | 0.5 |
| N05BB | Hydroxyzine | LD. Compatible in small doses. Larger doses or prolonged use may cause drowsiness to the infant or decrease milk supply. Use alternative drugs. | L2 | 1 | 0.1 |
| L04AB | Infliximab | LD. Probably Compatible. | L3 | 4 | 0.5 |
| J07BB | Influenza virus vaccines | Compatible. | L1 | 2 | 0.2 |
| A10AE | Insulin | Compatible. | L2 | 3 | 0.4 |
| C07AG | Labetalol | LD. Probably Compatible. Use alternative drugs for preterm infants. | L2 | 2 | 0.2 |
| A06AD | Lactulose | Not classified. | L3 | 11 | 1.3 |
| N03AX | Lamotrigine | Compatible. | L2 | 2 | 0.2 |
| N03AX | Levetiracetam | Probably Compatible. Might reduce milk supply. | L2 | 1 | 0.1 |
| R06AE | Levocetirizine | LD. Probably Compatible. High doses may decrease milk supply. | L2 | 4 | 0.5 |
| H03AA | Levothyroxine | LD. Compatible. | L1 | 192 | 22.7 |
| N01BB | Lidocaine | Compatible. | L2 | 44 | 5.2 |
| M01AC | Lornoxicam | Not classified. | Not classified. | 59 | 7.0 |
| A12CC | Magnesium Aspartate | ND. Probably Compatible. | Not classified. | 21 | 2.5 |
| A12CC | Magnesium Oxide | ND. Probably Compatible. | Not classified. | 1 | 0.1 |
| N02AB | Meperidine | LD. Infant's sedation. Use alternative drugs. | L4 | 7 | 0.8 |
| J01DH | Meropenem | ND. Probably Compatible. | L3 | 1 | 0.1 |
| A07EC | Mesalamine | Not classified. | L3 | 1 | 0.1 |
| A10BA | Metformin | Compatible. Use with caution in newborn, preterm infants and those with renal impairment. | L1 | 5 | 0.6 |
| N07BC | Methadone | Compatible. | L2 | 1 | 0.1 |
| C02AB | Methyldopa | Compatible. | L2 | 15 | 1.8 |
| H02AB | Methylprednisolone | Compatible. | L2 | 3 | 0.4 |
| A03FA | Metochlopramide | Compatible. Avoid prolonged use because of side effects to mothers. Questionable if increases milk supply. | L2. Prolonged use (>4 weeks) may be accompanied by side effects to mothers. Increases milk supply when PRL levels are low. | 39 | 4.6 |
| C07AB | Metoprolol | Compatible. | L2 | 1 | 0.1 |
| J01XD | Metronidazole | Opinions vary. Avoid long term therapy. Avoid BF for 12-24h after last maternal dose. Use alternative drugs if possible. | L2 | 224 | 26.4 |
| A02BB | Misoprostol | Compatible. | L2 | 6 | 0.7 |
| C07AB | Nebivolol | ND. Use alternative drugs. | L3. Use alternative drugs. | 1 | 0.1 |
| C08CA | Nifedipine | Compatible. | L2 | 8 | 0.9 |
| J01XE | Nitrofurantoin | Avoid BF when infant <8 days or G6PD deficiency. Use alternative drugs. | L2. Avoid BF when infant <1 month or G6PD deficiency. | 2 | 0.2 |
| N05AH | Olanzapine | LD. Probably Compatible. | L2 | 1 | 0.1 |

| | | | | | |
|---|---|---|---|---|---|
| A02BC | Omeprazole | LD. Compatible. | L2 | 64 | 7.6 |
| A04AA | Ondasetron | LD. Compatible. | L2 | 2 | 0.2 |
| M03BC | Orphenadrine Citrate & Paracetamol | ND. Use alternative drugs. | L3. Use with caution. | 1 | 0.1 |
| J05AH | Oseltamivir | LD. Compatible. | L2 | 3 | 0.4 |
| N03AF | Oxcarbazepine | LD. Probably Compatible. Use with caution. | L3 | 1 | 0.1 |
| N02BE | Paracetamol | Compatible. | L1 | 737 | 87 |
| N06AB | Paroxetine | LD. Probably Compatible. | L2 | 1 | 0.1 |
| J01CAR | Piperacillin & Tazobactam | ND. Probably Compatible. | L2 | 4 | 0.5 |
| H02AB | Prednisolone | Probably Compatible. In high doses, avoid BF for 4 hours. High doses may suppress lactation. | L2 | 1 | 0.1 |
| C07AA | Propranolol | Compatible. | L2 | 1 | 0.1 |
| H03AB | Propylthiouracil | LD. Probably Compatible (low dosage limited to 450 mg/dl). If liver toxicity is suspected, the drug should be discontinued. | L2 | 2 | 0.2 |
| C09AA | Ramipril | ND. Use alternative drugs. | L3 | 2 | 0.2 |
| A02BA | Ranitidine | Use alternative drugs. Ranitidine spontaneously breaks down to a cancer-causing chemical. | L2 | 7 | 0.8 |
| L01FA | Rituximab | LD. Probably Compatible. Use with caution during BF. | L3 | 1 | 0.1 |
| C05 | Ruscus aculeatus whole, Hesperidine, Ascorbic acid | Not classified. | Not classified. | 1 | 0.1 |
| R03AK | Salbutamol-Albuterol Sulfate & Ipratropium Bromide | ND. Compatible. | L2 | 7 | 0.8 |
| A12CE | Selenium | Not classified. | L3 | 1 | 0.1 |
| N02CC | Sumatriptan | LD. Probably Compatible. Withholding BF in preterm infants might be helpful. | L3 | 2 | 0.2 |
| N02AX | Tramadole | Probably Compatible. Caution is recommended. | L3. Caution is recommended. | 114 | 13.5 |
| A05AA | Ursodeoxycholic Acid | Not classified. | L3 | 1 | 0.1 |
| J05AB | Valacyclovir | LD. Compatible. | L2 | 2 | 0.2 |
| N03AG | Valproic Acid | Probably Compatible. Monitor infant for side effects. | L4. Neurobehavioral complications. | 2 | 0.2 |
| B03BA | Vitamin B12 | Compatible. | L1 | 1 | 0.1 |
| R01AA | Xylometazoline | Not classified. | Not classified. | 3 | 0.4 |

BF: Breastfeeding, LD: Limited Data, ND: No Data, PRL: Prolactin

**Table S3**. Risk classification of medicines used during lactation according to Lactmed and Hale. (Attica/Greece 2020; N=796/847)

| ATC | Medication | Lactmed | Hale | N | % |
|---|---|---|---|---|---|
| B01AC | Acetylosalicylic Acid | Probably compatible (lowdose). | L2 | 5 | 0.6 |
| J05AB | Acyclovir | Compatible. | L2 | 1 | 0.1 |
| L04AB | Adalimubab | LD. Probably compatible. | L3 | 4 | 0.5 |
| N05BA | Alprazolam | LD. Probably Compatible in low dose and short-term use. Use alternative drugs. | L3 | 1 | 0.1 |
| A02AD | Aluminium Hydroxide & Magnesium Hydroxide | AH: Not classified.<br>MH: Compatible. | AH: Not classified.<br>MH: L1 | 2 | 0.2 |
| R05CB | Ambroxol | Not classified. | Not classified. | 1 | 0.1 |
| J01MB | Amikacin | Probably compatible. | L2 | 4 | 0.5 |
| C08CA | Amlodipine | LD. Probably compatible. | L3 | 1 | 0.1 |
| J01CA | Amoxicillin | LD. Compatible. | L1 | 11 | 1.3 |
| J01CAR | Amoxicillin & Clavulanic Acid | LD. Compatible. | L1 | 7 | 0.8 |
| J01CA | Ampicillin | Compatible. | L1 | 7 | 0.8 |
| L04AX | Azathioprine | Probably Compatible. Use with caution during BF. Avoiding BF for 4 hours markedly decreases drug levels in breast milk. | L3. Use with caution. Monitor infant for signs of immunosuppress ion. | 2 | 0.2 |
| J01FA | Azithromycin | Probably Compatible. Risk for infantile hypertrophic pyloric stenosis (?). | L2 | 2 | 0.2 |
| D07CC | Betamethasone &Gentamycine (Topical) | Compatible for short-term topical use. Avoid nipple area. | L3 | 2 | 0.2 |
| N05BA | Bromazepam | Not classified. | Not classified. | 1 | 0.1 |
| R05CB | Bromhexine | Not classified. | Not classified. | 2 | 0.2 |
| R01AD | Budesonide | Compatible. | L1 | 1 | 0.1 |
| A03D | Butylscopolamine&P aracetamole | Not classified. | Not classified. | 10 | 1.2 |
| G02CB | Cabergolin | ND. Probably Compatible. Suppresses Lactation – Avoid during lactation (Women treated with cabergoline for pituitary adenomas who become pregnant can breastfed their infants with no apparent risk of recurrence). | L3. Careful administration in patient with hyperprolactine mia. | 2 | 0.2 |
| A12A | Calcium Salt | Not classified. | L3 | 532 | 62.8 |
| J01DC | Cefaclor | LD. Compatible. | L1 | 59 | 7 |
| J01DC | Cefoxitin | Compatible. | L1 | 209 | 24.7 |
| J01DD | Ceftriaxone | LD. Compatible. | L1 | 19 | 2.2 |

| | | | | | |
|---|---|---|---|---|---|
| J01DC | Cefuroxime | LD. Probably compatible. | L2 | 317 | 37.4 |
| R06AE | Cetirizine | Compatible. High doses may decrease milk supply. | L2 | 1 | 0.1 |
| A02BA | Cimetidine | LD. Probably Compatible for infants >2 months—Use alternative drugs (potential for causing hepatic enzyme inhibition). | L1. Compatible for short-term use. Use alternative drugs if possible. | 3 | 0.4 |
| J01MA | Ciprofloxacin | Probably Compatible.Avoiding BF for 3-4 hours decreases the exposure. | L3. Use alternative drugs if possible. | 4 | 0.5 |
| J01FA | Clarithromycin | Probably Compatible. Risk for infantile hypertrophic pyloric stenosis (?). | L1 | 6 | 0.7 |
| J01FF | Clindamycin | Probably Compatible. Use alternative drugs if possible. | L2 | 2 | 0.2 |
| M01AB | Diclofenac | LD. Probably Compatible. | L2 | 634 | 74.9 |
| R01AD | Dexamethasone (Topical) | ND. Probably Compatible. | L3 | 3 | 0.4 |
| R06AB | Dimethindene | Not classified. | Not classified. | 3 | 0.4 |
| J01AA | Doxycycline | Compatible in short-term use. Avoid prolonged use. | L3. Avoid prolonged use >21 days (dental staining or decrease bone growth). | 1 | 0.1 |
| G03AA | Drospirenone&Ethinylestradiol | Not classified. | L3. May suppress lactation. | 3 | 0.4 |
| G02AB | Ergometrine or Ergonovin Maleate | Avoid in breastfeeding mothers. Lowers PRL, decreases BF rates. | L3. Avoid prolonged use, may suppress lactation. | 511 | 60.3 |
| J01FA | Erythromycin | Probably Compatible. Risk for infantile hypertrophic pyloric stenosis (?). | L3. Risk for infantile hypertrophic pyloric stenosis (?). | 2 | 0.2 |
| M01AH | Etoricoxib | Not classified. | Not classified. | 1 | 0.1 |
| N01AH | Fentanyl | LD. Probably Compatible (low dose). | L2 | 593 | 70 |
| G01AF | Fenticonazole | Not classified. | Not classified. | 2 | 0.2 |
| B03A | Ferrous Sulfate | Compatible. | L1 | 609 | 71.9 |
| H02AA | Fludrocortisone | Not classified. | L3 | 3 | 0.4 |
| B03BB | Folic Acid | Not classified. | L1 | 45 | 5.3 |
| V08CA | Gadobutrol | LD. Probably Compatible. Use alternative agents with published experience. | L3 | 1 | 0.1 |
| J01MB | Gentamicin | LD. Probably Compatible. | L2 | 15 | 1.8 |
| S01CA | Gentamicin & Dexamethasone | LD. Probably Compatible. | Not classified. | 1 | 0.1 |
| B01AB | Heparin (low molecular weight) | ND. Probably Compatible. | L2 | 442 | 52.2 |

| | | | | | |
|---|---|---|---|---|---|
| H02AB | Hydrocortisone | ND. Use alternative drugs. | L3. Low dosages, short-term use. | 10 | 1.2 |
| P01BA | Hydroxychloroquine | LD. Probably Compatible. | L2 | 2 | 0.2 |
| N05BB | Hydroxyzine | LD. Compatible in small doses. Larger doses or prolonged use may cause drowsiness to the infant or decrease milk supply. Use alternative drugs. | L2 | 1 | 0.1 |
| L04AB | Infliximab | LD. Probably Compatible. | L3 | 3 | 0.4 |
| J07BB | Influenza virus vaccines | Compatible. | L1 | 2 | 0.2 |
| A10AE | Insulin | Compatible. | L2 | 3 | 0.4 |
| C07AG | Labetalol | LD. Probably Compatible. Use alternative drugs for preterm infant. | L2 | 2 | 0.2 |
| A06AD | Lactulose | Not classified. | L3 | 11 | 1.3 |
| R06AE | Levocetirizine | LD. Probably Compatible. High doses may decrease milk supply. | L2 | 4 | 0.5 |
| H03AA | Levothyroxine | LD. Compatible. | L1 | 184 | 21.7 |
| N01BB | Lidocaine | Compatible. | L2 | 44 | 5.2 |
| M01AC | Lornoxicam | Not classified. | Not classified. | 59 | 7.0 |
| A12CC | Magnesium Aspartate | ND. Probably Compatible. | Not classified. | 20 | 2.4 |
| N02AB | Meperidine | LD. Infant's sedation. Use alternative drugs. | L4 | 6 | 0.7 |
| J01DH | Meropenem | ND. Probably Compatible. | L3 | 1 | 0.1 |
| A07EC | Mesalamine | Not classified. | L3 | 1 | 0.1 |
| A10BA | Metformin | Compatible. Use with caution in newborn, preterm infants and those with renal impairment. | L1 | 5 | 0.6 |
| C02AB | Methyldopa | Compatible. | L2 | 13 | 1.5 |
| H02AB | Methylprednisolone | Compatible. | L2 | 2 | 0.2 |
| A03FA | Metochlopramide | Compatible. Avoid prolonged use because of side effects to mothers. Questionable if increases milk supply. | L2. Prolonged use (>4 weeks) may be accompanied by side effects to mothers. Increases milk supply when PRL levels are low. | 37 | 4.4 |
| C07AB | Metoprolol | Compatible. | L2 | 1 | 0.1 |
| J01XD | Metronidazole | Opinions vary. Avoid long term therapy. Avoid BF for 12-24h after last maternal dose. Use alternative drugs if possible. | L2 | 204 | 24.1 |
| A02BB | Misoprostol | Compatible. | L2 | 6 | 0.7 |
| C08CA | Nifedipine | Compatible. | L2 | 7 | 0.8 |
| J01XE | Nitrofurantoin | Avoid in BF when infant <8 days or G6PD deficiency. Use alternative drugs. | L2. Avoid in BF when infant <1 month or G6PD deficiency. | 1 | 0.1 |

| | | | | | |
|---|---|---|---|---|---|
| A02BC | Omeprazole | LD. Compatible. | L2 | 59 | 7 |
| A04AA | Ondasetron | LD. Compatible. | L2 | 2 | 0.2 |
| M03BC | Orphenadrine Citrate & Paracetamol | ND. Use alternative drugs. | L3. Use with caution. | 1 | 0.1 |
| J05AH | Oseltamivir | LD. Compatible. | L2 | 1 | 0.1 |
| N02BE | Paracetamol | Compatible. | L1 | 695 | 82 |
| J01CAR | Piperacillin & Tazobactam | ND. Probably Compatible. | L2 | 3 | 0.4 |
| C07AA | Propranolol | Compatible. | L2 | 1 | 0.1 |
| H03AB | Propylthiouracil | LD. Probably Compatible (low dosage limited to 450 mg/dl). If liver toxicity is suspected, the drug should be discontinued. | L2 | 1 | 0.1 |
| C09AA | Ramipril | ND. Use alternative drugs. | L3 | 2 | 0.2 |
| A02BA | Ranitidine | Use alternative drugs. Ranitidine spontaneously breaks down to a cancer-causing chemical. | L2 | 7 | 0.8 |
| C05 | Ruscus aculeatus whole, Hesperidine, Ascorbic acid | Not classified. | Not classified. | 1 | 0.1 |
| R03AK | Salbutamol-Albuterol Sulfate & Ipratropium Bromide | ND. Compatible. | L2 | 5 | 0.6 |
| A12CE | Selenium | Not classified. | L3 | 1 | 0.1 |
| N02CC | Sumatriptan | LD. Probably Compatible. Withholding breastfeeding in preterm infants might be helpful. | L3 | 1 | 0.1 |
| N02AX | Tramadole | Probably Compatible. Caution is recommended. | L3. Caution is recommended. | 106 | 12.5 |
| J05AB | Valacyclovir | LD. Compatible. | L2 | 1 | 0.1 |
| B03BA | Vitamin B12 | Compatible. | L1 | 1 | 0.1 |
| R01AA | Xylometazoline | Not classified. | Not classified. | 3 | 0.4 |

BF: Breastfeeding, LD: Limited Data, ND: No Data, PRL: Prolactin

**Table S4**: Cases of breastfeeding cessation due to medication intake and professional counseling. (Attica/Greece 2020; N= 57/847)

| Case | BF duration | Reason for Medication Intake | Medicines | Compatibility with breastfeeding | Recommendation and Decision for BF cessation | Counseling |
|---|---|---|---|---|---|---|
| 1 | 4 days | Depression | Escitalopram | **Lactmed**: LD. Probably Compatible.<br>**Hale**: L2 | Pediatrician recommended continuation of BF. Maternal decision.* | Evidence-based counseling. |
| 2 | 0 days | Depression | Escitalopram<br><br>Alprazolam | **Lactmed**: LD. Probably Compatible.<br>**Hale**: L2<br><br>**Lactmed**: LD. Probably Compatible in low dose and short-term use. Use alternative drugs (e.g., midazolam, oxazepam).<br>**Hale**: L3 | Obstetrician's advice. Informed choice.** | Erroneous counseling. The HP could have advised the mother to take measures to minimize the infant's exposure. |
| 3 | 15 days | Depression | Escitalopram | **Lactmed**: LD. Probably Compatible.<br>**Hale**: L2 | Pediatrician recommended continuation of BF. Maternal decision. | Evidence-based counseling. |
| 4 | 0 days | Depression | Escitalopram | **Lactmed**: LD. Probably Compatible.<br>**Hale**: L2 | Neurologist recommended continuation of BF. Maternal decision. | Evidence-based counseling. |
| 5 | 0 days | Tachycardia<br>Depression | Nebivolol<br><br>Bromazepam | **Lactmed**: ND. Use alternative drugs.<br>**Hale**: L3. Use alternative drugs (e.g., metoprolol, labetalol).<br><br>**Lactmed**: Not classified.<br>**Hale**: Not classified.<br>Note: Bromazepam's pharmacokinetic data makes it possible to pass into breast milk in significant amounts. Use alternative drugs (e.g., lormetazepam, temazepam) [37]. | Obstetrician's advice. Informed choice. | Erroneous counseling. The HP could have advised the mother to take measures to minimize the infant's exposure. (e.g., milk bank). |
| 6 | 20 days | Postpartum Depression | Fluoxetine | **Lactmed**: LD. Probably Compatible. Use alternative drugs if possible (e.g., sertraline, escitalopram).<br>**Hale**: L2. Use alternative drugs if possible. | Neurologist's advice. Obstetrician and Pediatrician in accordance. Informed choice. | Erroneous counseling. The HP could have advised the mother to take measures to minimize the infant's exposure. [e.g., use the medicine when breastfeeding older |

| | | | | | | |
|---|---|---|---|---|---|---|
| | | | | | | infants (4-6 months of age)]. |
| 7 | 60 days | Postapartum depression | Paroxetine | **Lactmed**: LD. Probably Compatible. **Hale**: L2 | Pediatrician's advice (IBCLC)/ [Infant neutropenia]. Informed choice. | Evidence-based counseling due to the onset of adverse side effects in the infant. |
| 8 | 40 days | Postpartum depression | Aripiprazole | **Lactmed**: LD. Probably Compatible in low doses. Use alternative drugs if possible (e.g., olanzapine, risperidone). May suppress lactation. **Hale**: L3. May suppress lactation. | Psychiatrist's advice. Informed choice. | Erroneous counseling. The HP could have advised the mother to take measures to minimize the infant's exposure. |
| 9 | 60 days | Postpartum depression | Olanzapine<br><br>Citalopram | **Lactmed**: LD. Probably Compatible **Hale**: L2<br><br>**Lactmed:** LD. Probably Compatible. Use alternative drugs if possible (e.g., sertraline, escitalopram). **Hale:** L2. Use alternative drugs if possible. | Neurologist's advice. Informed choice. | Erroneous counseling. The HP could have advised the mother to take measures to minimize the infant's exposure. |
| 10 | 0 days | Generalized anxiety disorder | Escitalopram | **Lactmed**: LD. Probably Compatible. **Hale**: L2 | Neurologist's advice. Informed choice. | Erroneous counseling due to compatibility of the medicine with BF. |
| 11 | 7 days | Panic disorder | Escitalopram | **Lactmed**: LD. Probably Compatible. **Hale**: L2 | Neurologist's advice. Informed choice. | Erroneous counseling due to compatibility of the medicine with BF. |
| 12 | 0 days | Bipolar disorder | Aripiprazole | **Lactmed**: LD. Probably Compatible in low doses. Use alternative drugs if possible (e.g., olanzapine, risperidone). May suppress lactation. **Hale**: L3. May suppress lactation. | Neurologist's advice. Obstetrician in accordance. Informed choice. | Erroneous counseling. The HP could have advised the mother to take measures to minimize the infant's exposure. |
| 13 | 0 days | Epilepsy | Carbamazepine | **Lactmed**: LD. Probably compatible. **Hale**: L2 | Neurologist's advice. Obstetrician in accordance. Informed choice. | Erroneous counseling due to compatibility of the medicine with BF. |
| 14 | 0 days | Epilepsy | Valproic Acid | **Lactmed**: Probably Compatible. Monitor infant for side effects. | Neurologist's/ Obstetrician's advice. Informed choice. | Evidence-based counseling. |

| | | | | | | |
|---|---|---|---|---|---|---|
| | | | | **Hale**: L4. Neurobehavioral complications. | | |
| 15 | 0 days | Epilepsy | Lamotrigine<br><br>Clonazepam | **Lactmed**: Compatible. **Hale**: L2<br><br>**Lactmed**: Compatible to use cautiously. Use alternative drugs if possible (long half-life) (e.g., carbamazepine). **Hale**: L3 | Neurologist's advice. Obstetrician and Pediatrician in accordance. Informed choice. | Erroneous counseling. The HP could have advised the mother to take measures to minimize the infant's exposure. |
| 16 | 0 days | Epilepsy | Lamotrigine<br><br>Levetiracetam | **Lactmed**: Compatible **Hale**: L2<br><br>**Lactmed**: Probably Compatible. Might reduce milk supply. **Hale**: L2 | Obstetrician's advice. Informed choice. | Erroneous counseling due to compatibility of the medicines with BF. |
| 17 | 0 days | Epilepsy | Oxcarbazepine | **Lactmed**: LD. Probably Compatible. Use with caution (Alternatives: e.g., carbamazepine). **Hale**: L3 | Neurologist's advice. Obstetrician in accordance. Informed choice. | Erroneous counseling. The HP could have advised the mother to take measures to minimize the infant's exposure. |
| 18 | 0 days | Multiple Sclerosis | Dimethyl fumarate | **Lactmed**: LD. Probably Compatible. **Hale**: L2 | Neurologist's advice. Informed choice. | Erroneous counseling due to compatibility of the medicine with BF. |
| 19 | 0 days | Multiple Sclerosis | Fingolimod | **Lactmed**: Avoid during breastfeeding. Potential toxicity to the infant. **Hale:** L5 | Neurologist's advice. Informed choice. | Evidence-based counseling. |
| 20 | 0 days | Multiple Sclerosis | Fingolimod | **Lactmed**: Avoid during breastfeeding. Potential toxicity to the infant. **Hale:** L5 | Neurologist's/ Obstetrician's advice. Informed choice. | Evidence-based counseling. |
| 21 | 180 days | Multiple sclerosis | Alemtuzumab | **Lactmed**: ND. Use with caution or avoid, especially while nursing a newborn or preterm infant. **Hale**: L4 | Neurologist's advice. Obstetrician in accordance. Informed choice. [Delayed the therapy in order to breastfeed] | Evidence-based counseling. |
| 22 | 150 days | Multiple sclerosis | Dimethyl fumarate | **Lactmed**: LD. Probably Compatible **Hale**: L2 | Neurologist's advice. Informed choice. | Erroneous counseling due to compatibility of the medicine with BF. |
| 23 | 0 days | Crohn's disease | Azathioprine | **Lactmed**: Probably Compatible. Use with caution during BF | Gastroenterologist's/Obstetrician's/Pediatrician's advice. | Erroneous counseling. The HP could have advised |

| | | | | | | |
|---|---|---|---|---|---|---|
| | | | | (Alternatives: e.g., infliximab, budesonide). Avoiding BF for 4 hours markedly decreases drug levels in breast milk.<br>**Hale**: L3 | Informed choice. | the mother to take measures to minimize the infant's exposure. [e.g., administer the maternal dose after BF and before the infant's longest sleep; avoid BF or discard breast milk for a certain period of time (e.g., 4 hours) after the maternal dose]. |
| 24 | 3 days | Sjorgen syndrome | Hydroxychloroquine | **Lactmed**: LD. Probably Compatible.<br>**Hale**: L2 | Rheumatologist's advice.<br>Obstetrician/ Pediatrician in accordance.<br>Informed choice. | Erroneous counseling due to compatibility of the medicine with BF. |
| 25 | 0 days | Systemic lupus erythematosus | Hydroxychloroquine<br><br>Methylprednisolone (8 mg/day) | **Lactmed**: LD. Probably Compatible<br>**Hale**: L2<br><br>**Lactmed**: Compatible<br>**Hale**: L2 | Obstetrician/pediatrician informed her that high doses of cortisone are incompatible with BF.<br>Maternal decision. | Erroneous counseling due to drug dose compatibility with BF. |
| 26 | 30 days | Cushing disease | Fludrocortisone<br><br>Hydrocortisone (6.7mg/day)<br><br>Magnesium Oxide | **Lactmed**: Not classified.<br>**Hale**: L3<br><br>**Lactmed**: ND. Use alternative drugs.<br>**Hale**: L3. Low dosages, short-term use.<br><br>**Lactmed**: ND. Probably Compatible.<br>**Hale**: Not classified. | Obstetrician's advice.<br>Informed choice. | Erroneous counseling. The HP could have advised the mother to take measures to minimize the infant's exposure. [e.g., administer the maternal dose after BF and before the infant's longest sleep; avoid BF or discard breast milk for a certain period of time (e.g., 4 hours) after the maternal dose]. |
| 27 | 135 days | Diabetes | Metformin | **Lactmed**: Compatible. Use with caution in newborn, preterm infants and those with renal impairment.<br>**Hale**: L1 | Endocrinologist's advice.<br>Informed choice. | Erroneous counseling due to compatibility of the medicine with BF. |
| 28 | 0 days | Hyperthyroidism | Propylthiouracil | **Lactmed**: LD. Probably Compatible (low dosage | Endocrinologist's advice. | Erroneous counseling due to |

| | | | | | | |
|---|---|---|---|---|---|---|
| | | | | limited to 450 mg/dl). If liver toxicity is suspected, the drug should be discontinued. **Hale**: L2 | Obstetrician in accordance. Informed choice. | compatibility of the medicine with BF. |
| 29 | 20 days | Mastitis | Amoxicillin & Clavulanic Acid<br><br>Diclofenac | **Lactmed**: LD. Compatible. **Hale**: L1<br><br>**Lactmed**: LD. Probably Compatible. **Hale**: L2 | Obstetrician's advice to discard breast milk. Maternal decision to cease due to milk reduction. | Erroneous counseling due to compatibility of the medicines with BF. |
| 30 | 7 days | Mastitis | Amoxicillin & Clavulanic Acid<br><br>Metronidazole | **Lactmed**: LD. Compatible. **Hale**: L1<br><br>**Lactmed**: Opinions vary. Avoid long term therapy. Avoid BF for 12-24h after last maternal dose. Use alternative drugs if possible. **Hale**: L2 | Obstetrician's advice. Informed decision. | Erroneous counseling. The HP could have advised the mother to take measures to minimize the infant's exposure. |
| 31 | 40 days | Mastitis | Cefaclor<br><br>Ciprofloxacin | **Lactmed**: LD. Compatible. **Hale**: L1<br><br>**Lactmed**: Probably Compatible. Avoiding BF for 3-4 hours decreases the exposure. **Hale**: L3. Use alternative drugs if possible (e.g., amoxicilline, cephalexin, ofloxacin). | Mammologist's advice. Informed choice. | Erroneous counseling. The HP could have advised the mother to take measures to minimize the infant's exposure. [e.g., administer the maternal dose after BF and before the infant's longest sleep; avoid BF or discard breast milk for a certain period of time (e.g., 4 hours) after the maternal dose]. |
| 32 | 30 days | Postpartum infection | Cefoxitin<br><br>Metronidazole | **Lactmed**: Compatible. **Hale**: L1<br><br>**Lactmed**: Opinions vary. Avoid long term therapy. Avoid BF for 12-24h after last maternal dose. Use alternative drugs if possible. **Hale**: L2 | Obstetrician's advice. Informed choice. | Erroneous counseling. The HP could have advised the mother to take measures to minimize the infant's exposure. |

| | | | | | | |
|---|---|---|---|---|---|---|
| | | | Doxycycline (10 days) | **Lactmed**: Compatible in short-term use. Avoid prolonged use.<br>**Hale**: L3. Avoid prolonged use >21 days (dental staining or decrease bone growth). | | |
| 33 | 4 days | Postpartum infection | Cefuroxime | **Lactmed**: LD. Probably compatible.<br>**Hale**: L2 | Obstetrician's advice. Informed decision.<br><br>The pediatrician recommended her relactation, but the mother refused. | Erroneous counseling. The HP could have advised the mother to take measures to minimize the infant's exposure. |
| | | | Metronidazole | **Lactmed**: Opinions vary. Avoid long term therapy. Avoid BF for 12-24h after last maternal dose. Use alternative drugs if possible.<br>**Hale**: L2 | | |
| | | | Doxycycline (10 days) | **Lactmed**: Compatible in short-term use. Avoid prolonged use.<br>**Hale**: L3. Avoid prolonged use >21 days (dental staining or decrease bone growth). | | |
| 34 | 40 days | Endometritis | Cefuroxime | **Lactmed**: LD. Probably compatible.<br>**Hale**: L2 | Obstetrician's advice. Informed choice. | Erroneous counseling. The HP could have advised the mother to take measures to minimize the infant's exposure. |
| | | | Metronidazole | **Lactmed**: Opinions vary. Avoid long term therapy. Avoid BF for 12-24h after last maternal dose. Use alternative drugs if possible.<br>**Hale**: L2 | | |
| | | | Amikacin | **Lactmed**: Probably compatible.<br>**Hale**: L2 | | |
| 35 | 0 days | Respiratory tract infection | Ceftriaxone<br><br>Azithromycin<br><br>Salbutamol-Albuterol Sulfate | **Lactmed**: LD. Compatible.<br>**Hale**: L1<br><br>**Lactmed**: Probably Compatible. Risk for infantile hypertrophic pyloric stenosis (?).<br>**Hale**: L2 | Obstetrician's advice. Informed choice. | Erroneous counseling due to compatibility of the medicines with BF.<br><br>Note: Ambroxol is a mucolytic of choice, widely used and well tolerated during BF [37]. |

| | | | | | | |
|---|---|---|---|---|---|---|
| | | | & Ipratropium Bromide<br><br>Budesonide<br><br>Ambroxol | **Lactmed**: ND. Compatible.<br>**Hale**: L2<br><br>**Lactmed**: Compatible.<br>**Hale**: L1<br><br>**Lactmed:** Not classified.<br>**Hale**: Not classified. | | |
| 36 | 15 days | Pyelonephritis | Ciprofloxacin | **Lactmed**: Probably Compatible. Avoiding BF for 3-4 hours decreases the exposure.<br>**Hale**: L3. Use alternative drugs if possible (e.g., amoxicilline, cephalexin, ofloxacin). | Obstetrician's/ Pediatrician's advice. Informed choice. | Erroneous counseling. The HP could have advised the mother to take measures to minimize the infant's exposure. [e.g., administer the maternal dose after BF and before the infant's longest sleep; avoid BF or discard breast milk for a certain period of time (e.g., 4 hours) after the maternal dose]. |
| 37 | 60 days | Pyelonephritis | Ciprofloxacin<br><br>Amoxicillin & Clavulanic Acid<br><br>Cefoxitin | **Lactmed**: Probably Compatible. Avoiding BF for 3-4 hours decreases the exposure.<br>**Hale**: L3. Use alternative drugs if possible (e.g., amoxicilline, cephalexin, ofloxacin).<br><br>**Lactmed:** LD. Compatible<br>**Hale:** L1<br><br>**Lactmed:** Compatible<br>**Hale:** L1 | Obstetrician's advice. Informed choice. | Erroneous counseling. The HP could have advised the mother to take measures to minimize the infant's exposure. [e.g., administer the maternal dose after BF and before the infant's longest sleep; avoid BF or discard breast milk for a certain period of time (e.g., 4 hours) after the maternal dose]. |
| 38 | 4 days | Urinary tract infection | Ciprofloxacin | **Lactmed**: Probably Compatible. Avoiding BF for 3-4 hours decreases the exposure.<br>**Hale**: L3. Use alternative drugs if possible. | Obstetrician recommended discarding the breast milk. | Evidence-based counseling. |

| | | | | | | |
|---|---|---|---|---|---|---|
| | | | | | Maternal decision to cease due to milk reduction. | |
| 39 | 150 days | Conjunctivitis | Gentamicin & Dexamethasone (Topical) | **Lactmed:** LD. Probably Compatible. **Hale**: Not classified. | Pediatrician recommended discarding the breast milk. Maternal decision to cease BF due to milk reduction. | Erroneous counseling due to compatibility of the medicine with BF. |
| 40 | 60 days | Ulcerative colitis | Azathioprine | **Lactmed**: Probably Compatible. Use with caution during BF (Alternatives: e.g., infliximab, budesonide). Avoiding BF for 4 hours markedly decreases drug levels in breast milk. **Hale**: L3 | Gastroenterologist's advice. Pediatrician in accordance. Informed choice. | Erroneous counseling. The HP could have advised the mother to take measures to minimize the infant's exposure. [e.g., administer the maternal dose after BF and before the infant's longest sleep; avoid BF or discard breast milk for a certain period of time (e.g., 4 hours) after the maternal dose]. |
| | | | Infliximab | **Lactmed**: LD. Probably Compatible. **Hale**: L3 | | |
| | | | Prednisolone (40mg/day) | **Lactmed**: Probably Compatible. In high doses (>40 mg/day), avoid BF for 4 hours. High doses may suppress lactation. **Hale**: L2 | | |
| 41 | 20 days | Purulent drainage from wounds | Ciprofloxacin | **Lactmed**: Probably Compatible. Avoiding BF for 3-4 hours decreases the exposure. **Hale**: L3. Use alternative drugs if possible. | Obstetrician's advice to discard breast milk. | Evidence-based counseling. |
| | | | Metronidazole | **Lactmed**: Opinions vary. Avoid long term therapy. Avoid BF for 12-24h after last maternal dose. Use alternative drugs if possible. **Hale**: L2 | Maternal decision. | |
| | | | Amoxicillin | **Lactmed**: LD. Compatible. **Hale**: L1 | | |
| 42 | 9 days | Purulent drainage | Clindamycin | **Lactmed:** Probably Compatible. Use | Maternal decision. | Absence of counseling. |

| | | | | | | |
|---|---|---|---|---|---|---|
| | | from wounds | | alternative drugs if possible.<br>**Hale:** L2. LD. Probably Compatible. | | |
| | | | Doxycycline | **Lactmed**: Compatible in short-term use. Avoid prolonged use.<br>**Hale**: L3. Avoid prolonged use >21 days (dental staining or decrease bone growth). | | |
| | | | Piperacillin & Tazobactam | **Lactmed**: ND. Probably Compatible.<br>**Hale**: L2 | | |
| 43 | 10 days | Purulent drainage from wounds | Clindamycin | **Lactmed:** Probably Compatible. Use alternative drugs if possible.<br>**Hale:** L2. LD. Probably Compatible. | Pediatrician recommended discarding the breast milk.<br>Maternal decision to cease BF due to milk reduction. | Evidence-based counseling. |
| | | | Ciproflocxacin | **Lactmed**: Probably Compatible. Avoiding BF for 3-4 hours decreases the exposure.<br>**Hale:** L3. Use alternative drugs if possible. | | |
| 44 | 60 days | Purulent tonsillitis, allergy to antibiotic | Methylprednisolone (12 mg/day) | **Lactmed**: Compatible.<br>**Hale**: L2 | Maternal decision (internet information). | Absence of counseling. |
| 45 | 165 days | Influenza | Oseltamivir | **Lactmed**: LD. Compatible.<br>**Hale**: L2 | Pathologist's and obstetrician's advice. Informed choice. | Erroneous counseling due to compatibility of the medicines with BF. |
| | | | Clarithromycin | **Lactmed**: Probably Compatible. Risk for infantile hypertrophic pyloric stenosis (?).<br>**Hale**: L1 | | |
| 46 | 5 days | Prophylactic administration for influenza (older's child illness) | Oseltamivir | **Lactmed**: LD. Compatible.<br>**Hale**: L2 | Pediatrician's advice. Informed choice. | Erroneous counseling due to compatibility of the medicine with BF. |
| 47 | 45 days | Herpes zoster | Valacyclovir | **Lactmed**: LD. Compatible. | Obstetrician's/ Pediatrician's advice. | Erroneous counseling due to |

| | | | | | | |
|---|---|---|---|---|---|---|
| | | | | **Hale**: L2 | Informed choice. | compatibility of the medicine with BF. |
| 48 | 4 days | Hypertension | Methyldopa<br><br>Nifedipine | **Lactmed**: Compatible.<br>**Hale**: L2<br><br>**Lactmed**: Compatible.<br>**Hale**: L2 | Pediatrician recommended continuation of BF. Maternal decision. | Evidence-based counseling. |
| 49 | 60 days | Hypertension | Methyldopa | **Lactmed**: Compatible<br>**Hale**: L2 | Obstetrician/Paediatrician recommended discarding the breast milk.<br>Maternal decision to cease BF due to milk reduction. | Erroneous counseling due to compatibility of the medicine with BF. |
| 50 | 135 days | Cholestasis | Ursodeoxycholic Acid | **Lactmed**: Not classified<br>**Hale**: L3 | Surgeon's/Pathologist's advice.<br>Informed choice, (fear of harming her infant, although internet information indicated compatibility of the medicine). | Erroneous counseling. The HP could have advised the mother to take measures to minimize the infant's exposure. (e.g., administer the maternal dose after BF and before the infant's longest sleep). |
| 51 | 40 days | Cholocystectomy | Ciprofloxacin<br><br>Metronidazole<br><br>Amoxicillin | **Lactmed**: Probably Compatible. Avoiding BF for 3-4 hours decreases the exposure (e.g., amoxicilline, cephalexin, ofloxacin).<br>**Hale**: L3. Use alternative drugs if possible.<br><br>**Lactmed**: Opinions vary. Avoid long term therapy. Avoid BF for 12-24h after last maternal dose. Use alternative drugs if possible.<br>**Hale**: L2<br><br>**Lactmed**: LD. Compatible.<br>**Hale**: L1 | Surgeon's advice. Informed choice. | Erroneous counseling. The HP could have advised the mother to take measures to minimize the infant's exposure. [e.g., administer the maternal dose after BF and before the infant's longest sleep; avoid BF or discard breast milk for a certain period of time (e.g., 4 hours) after the maternal dose]. |
| 52 | 150 days | Car accident-Hospitalization | Medicines that the mother cannot remember (included antiepileptics) | | Surgeon's/ Pathologist's advice. Informed choice. | Ambiguous recommendations due to inadequate information given about which |

| | | | | | | |
|---|---|---|---|---|---|---|
| | | | | | | medicines were administered. |
| 53 | 40 days | Kidney colic/ Nephrolithiasis | Alfuzosin | **Lactmed**: Not classified. **Hale**: L4 | Pathologist's/ Obstetrician's advice. Pediatrician in accordance. Informed choice. | Evidence-based counseling. |
| 54 | 90 days | Hypercholesterol emia | Atorvastatin | **Lactmed**: ND. Use alternative drugs (e.g., Cholestyramine, Colesevelam). **Hale**: L3 | Pathologist's advice. Informed choice. | Erroneous counseling. The HP could have advised the mother to take measures to minimize the infant's exposure. (e.g., milk bank) |
| 55 | 0 days | Thrombophilia | Acetylosalicylic Acid (160mg/day) | **Lactmed**: Probably compatible (low-dose) **Hale**: L2 | Haematologist's advice. Informed choice. | Erroneous counseling due to drug dose compatibility with BF. |
| 56 | 0 days | Illicit drug abuse/ former heroin user | Methadone | **Lactmed**: Compatible. **Hale**: L2 | Maternal decision. | Absence of counseling. |
| 57 | 0 days | Illicit drug abuse/ former heroin user | Buprenorphine & Naloxone | **Lactmed**: LD. Probably Compatible. **Hale**: L2 | Maternal decision. | Absence of counseling. |

BF: Breastfeeding, LD: Limited Data, ND: No Data, PRL: Prolactin.
* Maternal decision: the mother decides to cease breastfeeding in the absence of or contradictory to counseling. Maternal decision to cease breastfeeding due to milk reduction.
** Informed choice: the mother agrees and follows the HP's counseling for breastfeeding cessation.
